# GERIATRIC PRACTICE LEADERSHIP INSTITUTE—AN INTERPROFESSIONAL TEAM APPROACH USING THE IHI AGE-FRIENDLY MODEL

**DOI:** 10.1093/geroni/igac059.132

**Published:** 2022-12-20

**Authors:** Janice Knebl, Jennifer Severance, Sara Murphy, Kathlene Camp, James Roach, Thomas Fairchild

**Affiliations:** University of North Texas Health Science Center-TCOM, Fort Worth, Texas, United States; UNTHSC-TCOM, Fort Worth, Texas, United States; UNTHSC-TCOM, Fort Worth, Texas, United States; UNTHSC-TCOM, Fort Worth, Texas, United States; TCU - Neeley School of Business, Fort Worth, Texas, United States; UNTHSC, Fort Worth, Texas, United States

## Abstract

According to the Institute of Medicine, immediate steps must be taken to educate and train both the current and future health care workforce to work collaboratively in addressing the diverse needs of the growing older adult population.1 Most healthcare professionals had very little education or clinical training in the care of older adults nor the most effective ways to work as a clinical team. The Geriatric Practice Leadership Institute (GPLI) is a collaboration between two universities providing inter-professional teams of early and mid-career professionals the skills and knowledge needed to leverage leadership skills to effectively work within interdisciplinary teams to provide age-friendly care to older adults. The GPLI incorporates the Institute for Healthcare Improvement (IHI) Age-Friendly Health Systems 4Ms’ Framework into the training. The GPLI is an on-line, team-based program which engages 5-7 teams each session. Module topics include Age-Friendly Health Systems, organizational culture, leading self, leading inter-professional teams, and quality improvement. Additionally, teams select and completes a quality improvement project based on the Age-Friendly Health Systems 4Ms and submits a final report and presentation. The teams are also assigned a coach for support. Continuing education credits and a micro-credential are available to participants. The GPLI has trained over 175 health care professionals during the past 7 years with teams representing ambulatory to emergency responder organizations. The GPLI has been funded by the Health Resources and Services Administration (HRSA) Geriatrics Workforce Enhancement Program grant (numberU1QHP2873), which currently covers all costs for participants.

